# Tissue registration and exploration user interfaces in support of a human reference atlas

**DOI:** 10.1038/s42003-022-03644-x

**Published:** 2022-12-13

**Authors:** Katy Börner, Andreas Bueckle, Bruce W. Herr, Leonard E. Cross, Ellen M. Quardokus, Elizabeth G. Record, Yingnan Ju, Jonathan C. Silverstein, Kristen M. Browne, Sanjay Jain, Clive H. Wasserfall, Marda L. Jorgensen, Jeffrey M. Spraggins, N. Heath Patterson, Griffin M. Weber

**Affiliations:** 1 Department of Intelligent Systems Engineering, Luddy School of Informatics, Computing, and Engineering, Indiana University, Bloomington, IN USA; 2 Department of Biomedical Informatics, University of Pittsburgh School of Medicine, Pittsburgh, PA USA; 3 Bioinformatics and Computational Biosciences Branch, Office of Cyber Infrastructure and Computational Biology, National Institute of Allergy and Infectious Diseases, National Institutes of Health, Bethesda, MD USA; 4 Department of Medicine, Department of Pathology & Immunology, Department of Pediatrics, Washington University School of Medicine, Saint Louis, MO USA; 5 Departments of Pathology and Pediatrics, University of Florida, Gainesville, FL USA; 6 Mass Spectrometry Research Center, Vanderbilt University, Nashville, TN USA; 7 Department of Biomedical Informatics, Harvard Medical School, Boston, MA USA

**Keywords:** Software, Data integration

## Abstract

Seventeen international consortia are collaborating on a human reference atlas (HRA), a comprehensive, high-resolution, three-dimensional atlas of all the cells in the healthy human body. Laboratories around the world are collecting tissue specimens from donors varying in sex, age, ethnicity, and body mass index. However, harmonizing tissue data across 25 organs and more than 15 bulk and spatial single-cell assay types poses challenges. Here, we present software tools and user interfaces developed to spatially and semantically annotate (“register”) and explore the tissue data and the evolving HRA. A key part of these tools is a common coordinate framework, providing standard terminologies and data structures for describing specimen, biological structure, and spatial data linked to existing ontologies. As of April 22, 2022, the “registration” user interface has been used to harmonize and publish data on 5,909 tissue blocks collected by the Human Biomolecular Atlas Program (HuBMAP), the Stimulating Peripheral Activity to Relieve Conditions program (SPARC), the Human Cell Atlas (HCA), the Kidney Precision Medicine Project (KPMP), and the Genotype Tissue Expression project (GTEx). Further, 5,856 tissue sections were derived from 506 HuBMAP tissue blocks. The second “exploration” user interface enables consortia to evaluate data quality, explore tissue data spatially within the context of the HRA, and guide data acquisition. A companion website is at https://cns-iu.github.io/HRA-supporting-information/.

## Abstract (web summary)

The software tools and user interfaces for the registration and exploration of tissue data for the human reference atlas are presented, providing evaluation metrics for data quality examination and guidance for data acquisition in support of reference atlas construction.

## Introduction

The National Institutes of Health funded HuBMAP consortium was formed in 2018 to develop an open and global platform to map the estimated 37 trillion healthy cells in the human body^1,2^. An essential component of this platform are two software programs whose user interfaces enable organ experts to “register” tissue data and researchers to “explore” tissue data in a spatially and semantically explicit manner within the context of the evolving human atlas. The software programs are open and generalizable; and, they have been adopted by other international consortia mapping the human body. By combining and harmonizing datasets from these different consortia, the software is facilitating the collaborative construction of a consensus human reference atlas (HRA), which will be more accurate and more complete than would possible using data from only one consortium.

However, constructing an HRA that captures macro to microanatomy of healthy adults down to the single-cell level poses diverse challenges^3^. First, *specimen* metadata collected by different laboratories for diverse donors (e.g., basic demographics such as sex and age) and different assay types (e.g., spatial transcriptomics^4,5^ or CO-Detection by indEXing (CODEX)^6^ and associated data provenance) needs to be harmonized so they can be cross-searched. Second, *biological structure* data (i.e., information on what anatomical or histological structures and cell types are present in a tissue blocks extracted from human organs for interrogation) needs to be collected in a standard manner. Third, the *spatial* size of the tissue block, along with the position and rotation of the block in relation to reference organs positioned in a common three-dimensional (3D) reference space, need to be recorded.

To address these challenges, the registration and exploration user interfaces leverage a common coordinate framework (CCF), which is a “spatiotemporal computational framework for the management, integration, and analysis of anatomically and spatially indexed data”^7^. It creates “an underlying reference map of organs, tissues, or cells that allows new individual samples to be mapped to determine the relative location of structural regions between samples”^8^. In 2017, NIH organized a CCF meeting^9^ which reviewed different atlas construction projects. The Allen Mouse Brain Atlas^10^ was highlighted as a successful example of combining large-scale mapping, quantification, analysis, and visualization of data. Brain atlas projects identify anatomic-scale landmarks (e.g., specific brain folds, specific lumens with cerebrospinal fluid, or major arteries) that are conserved between individuals and apply mathematical coordinate mappings to register data from individual donor tissue samples to a reference geometry, allowing direct comparison across individuals. In particular, the Talairach and the Montreal Neurological Institute Hospital stereotaxic space emerged as CCFs for human brain tissue. For rodent brains, tThe Paxinos’ Rat brain stereotaxic coordinate system and Waxholm space^11,12^ are widely used^13–17^. However, a CCF that works for the brain^18^ does not necessarily work for other human organs that might be much larger (large intestine), in/deflated (lung), or highly variable in size (lymph nodes). Therefore, we constructed a CCF for the human reference atlas (called CCF-HRA) which combines expert-curated ontologies for specimen, biological structure, and spatial data^19^.

## Results

### Tissue registration user interface (RUI)

Groups contributing tissue data to advance the HRA need an effective means to register their data within a well-defined 3D spatial reference system and derive anatomical structure (AS) annotations. Specifically, they need to select the most appropriate reference organ (e.g., the male right kidney if this is the organ from which tissue was extracted) and specify the size, position, and rotation of a tissue block in relation to that organ. They must be able to review the listing of automatically assigned anatomical structure annotations and make changes as needed (add or delete annotations) to describe what anatomical regions the tissue block captures.

The Registration User Interface (RUI), available at https://hubmapconsortium.github.io/ccf-ui/rui, supports the registration of tissue data extracted either as tissue blocks or via biopsies. It allows RUI users to document the tissue extraction site in relation to a 3D reference organ. Note that RUI users have different background and expertise, e.g., the six individuals who collectively, registered more than 5000 tissue blocks comprise one computational biologist/clinician, one biomedical researcher/basic science researcher, one clinician, one basic science researcher, one biomedical/basic science/clinician, and one information scientist.

**Fig. 1:**
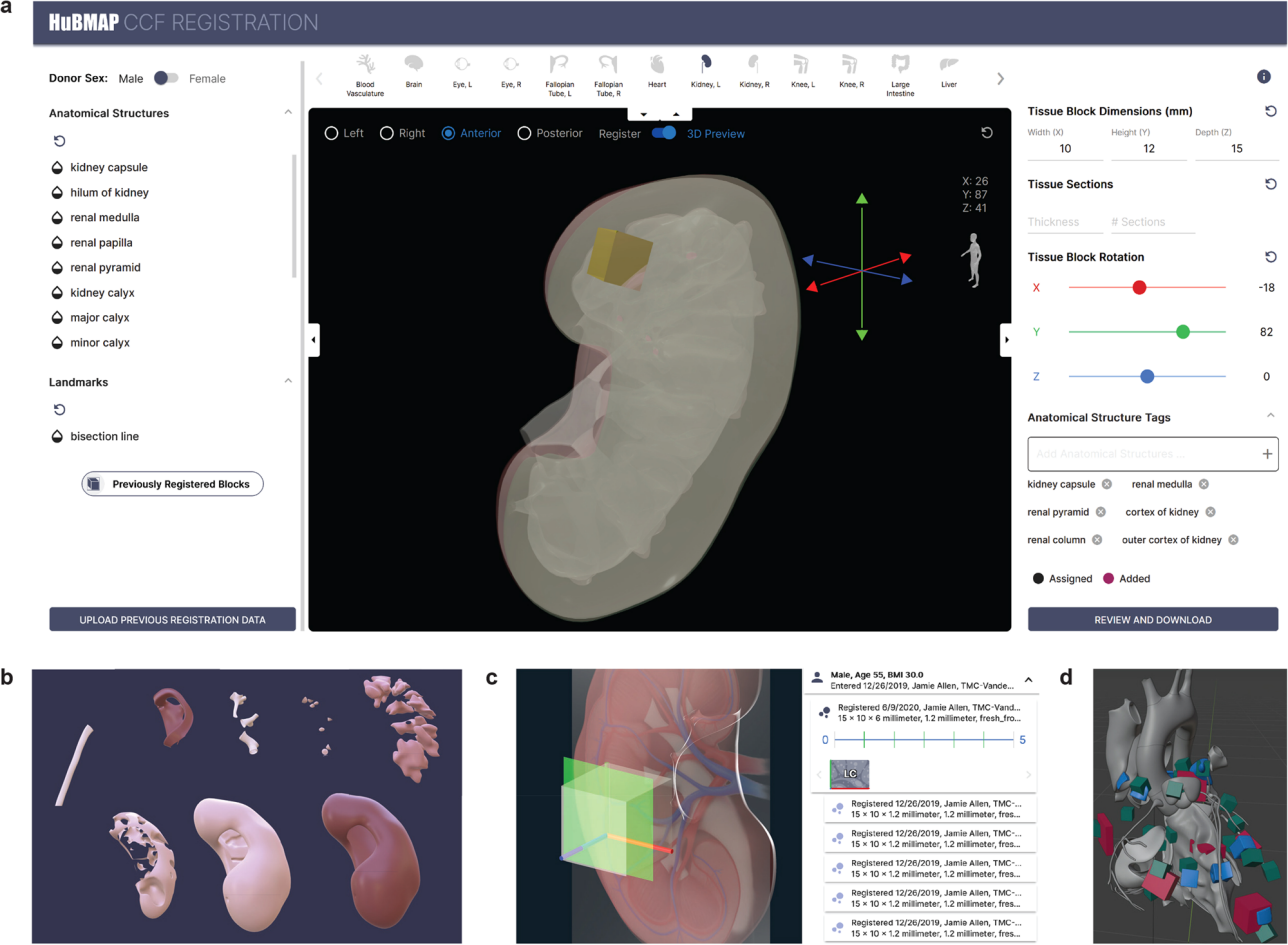
Tissue Registration User Interface (RUI). **a** Tissue registration for kidney using the RUI. **b** Named anatomical structures that together make up the male right kidney reference object. **c** Relationship of cutting plane (in green) to x-y-z coordinate system (red, green, blue axes) of the 3D tissue block are shown on left. Details panel on the right-hand side of the EUI with information on what tissue sections exist for a tissue block (blue lines in strip plot) and green-red axes on the left and bottom side of each thumbnail. **d** Common tissue extraction sites used by three different consortia for the male heart (six sites for HCA in red, 10 sites for HuBMAP in blue, 25 sites for SPARC in green).

The RUI web interface lets users enter metadata such as their name; next, they select an organ from the 3D Reference Object Library^20^. Using the 3D interface, they then define the size of a virtual tissue block and its position via drag-and-drop (in yellow in Fig. 1a) inside a reference organ. Collision detection (the intersection of bounding volumes detected by an algorithm; see anatomical structures that make up the kidney reference in Fig. 1b) is used to assign anatomical structure annotations (or “tags”, see lower right of Fig. 1a). If tissue sections are cut from a tissue block, it is important to keep track of the z-stack of tissue sections and their spatial relationships to the tissue block (see Fig. 1c). A user can define common tissue extraction sites (see Fig. 1d) and assign these to multiple tissue samples extracted from that same site (see Usage of the RUI and EUI section). For example, 138 GTEx tissue blocks were sampled from female donors between the ages of 21 and 70 years (mean: 53.2 years) from the very same extraction site in the left ventricle of the heart.

Some 3D reference objects have ‘Landmarks’ that help organ experts register tissue (e.g., see ‘bisection line’ for kidney in Fig. 1a). Previously registered tissue blocks can be made visible to guide the registration of new tissue blocks (e.g., to register blocks that were cut adjacent to an already registered tissue block).

### Tissue exploration user interface (EUI)

The Exploration User Interface (EUI), available at https://portal.hubmapconsortium.org/ccf-eui, was implemented for two types of stakeholders: Tissue data providers and tissue data and human reference atlas data users. (1) Tissue data providers can use the EUI to check and approve tissue size, location, rotation, and semantic annotations for the tissue blocks they registered with the RUI and the proper interlinkage of these tissue blocks with derivative datasets. RUI registrations are submitted via the HuBMAP Ingest Portal, processed on the backend, and then become available in the EUI for review and approval (see detailed steps in SOP: Using the CCF RUI ^20^). (2) Tissue and atlas data users come to the EUI to filter, browse, and search for tissue and atlas data using specimen, anatomical structures, or spatial data queries. Specifically, users expressed interest in running queries such as: What cell types are *located_in* which anatomical structure? What cell types/anatomical structures are in 1 mm distance from cell x? Which biomarkers *characterize* a certain cell type in a specific anatomical structure or functional tissue unit (FTU, defined as the smallest tissue organization that performs a unique physiologic function)? How many cells of type y are in anatomical structure of type x? How does the cell type population for the very same region (e.g., a set of anatomical structures) differ across sex, age, or ethnic groups? Plus, there is a strong interest in running spatial queries across donors or assay types, such as: For the same 1 mm^3^ in a reference body (e.g., the female body), what tissue blocks exist for a donor population (e.g., 25–30-year-old females) and/or a specific assay type (e.g., CODEX data)? Given a tissue block, what other tissue blocks exist for that same 3D volume?

**Fig. 2:**
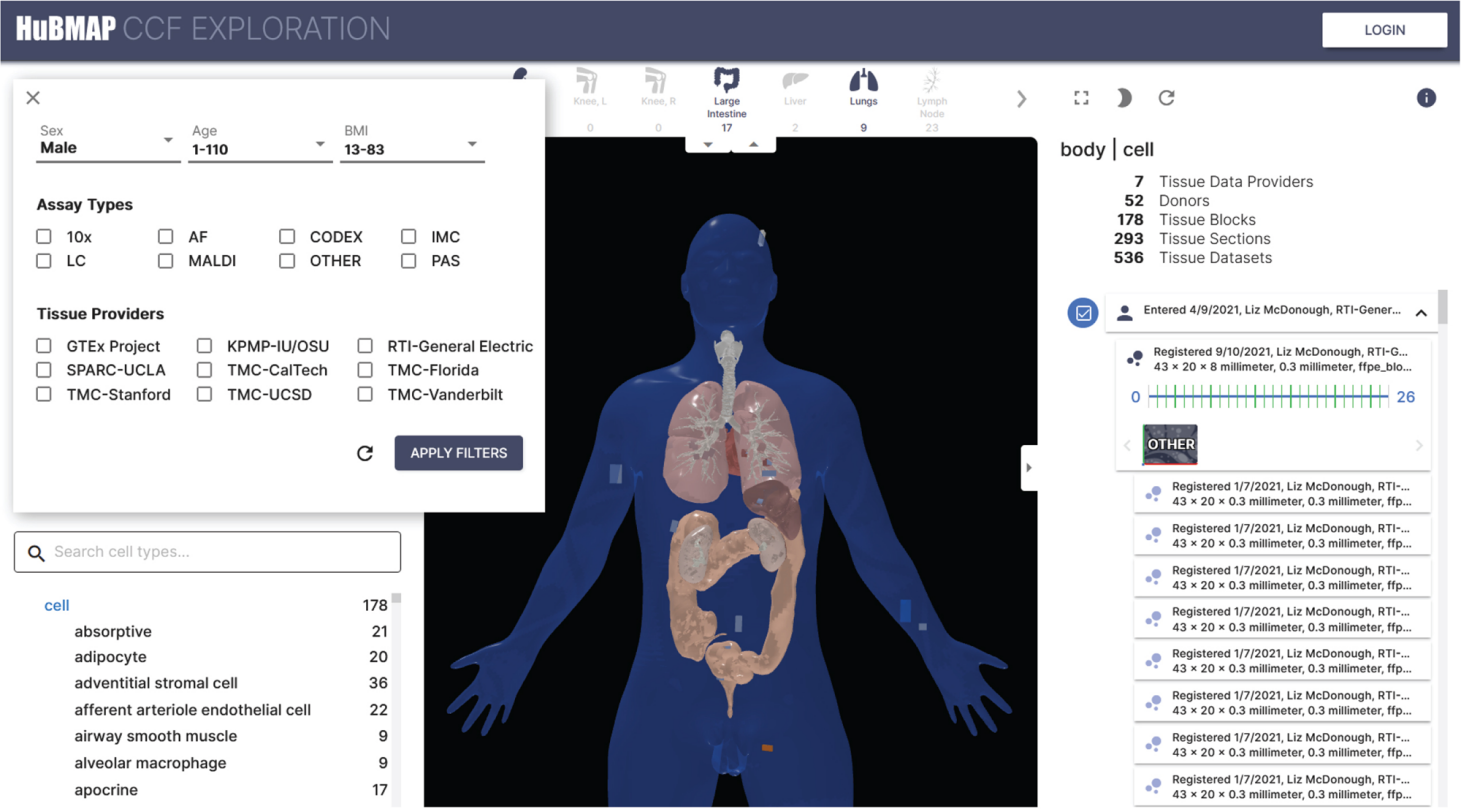
Tissue Exploration User Interface (EUI). The EUI features an anatomical structure “partonomy” on top left (occluded by filter menu, but see Fig. 3c) and a listing of unique cell types in lower left; 3D reference organs with registered tissue blocks (in white) and one selected skin tissue block in upper left leg (in orange) in middle; and details on all filtered tissue blocks, sections, and datasets on right–the selected skin tissue block has 26 tissue sections, each section has associated datasets. Filters for specimen metadata are provided in top left (sex, age, BMI, assay types, tissue providers). They are accessible via a filter menu; filter for sex has been set to 'Male.' Registered tissue blocks can be explored via specimen data, biological structure terms, or spatially using the 3D reference organ framework.

**Fig. 3:**
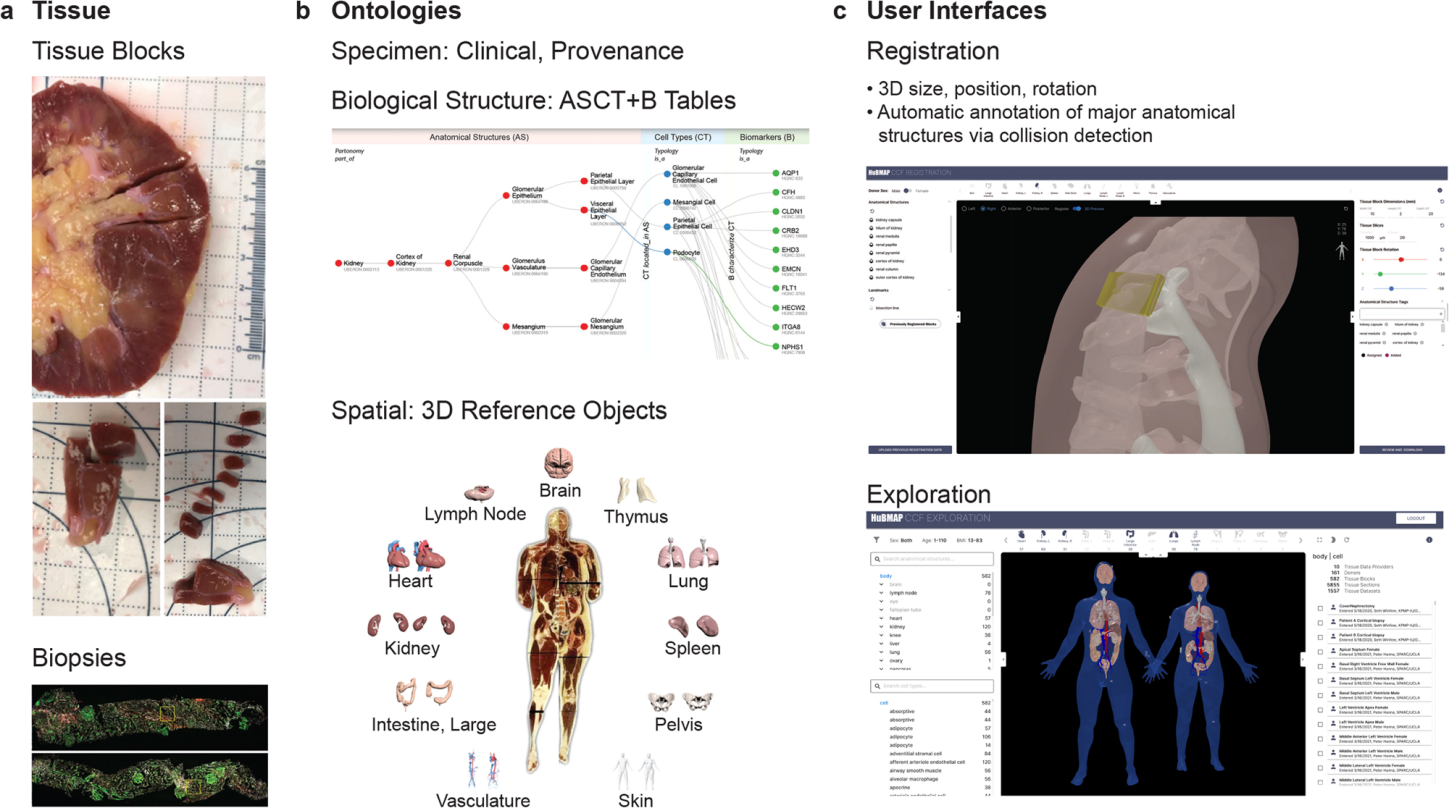
Workflow. **a** Tissue data (blocks or biopsies) are secured by different laboratories/projects from different donors and organs. **b** Three different ontologies capture specimen, biological structure, and spatial metadata derived from organ-specific ASCT+B tables and 3D reference objects. **c** The RUI (top) is used to register tissue data while the EUI (bottom) supports ontologically and spatially explicit exploration of tissue and HRA data across organs, donors, laboratories, and consortia.

The current EUI supports exploration of anatomical structures and cell types predicted via organ-specific ASCT+B tables^19^. It uses a split-screen interface (see Figs. 2 and 3c): A hierarchical list of anatomical structures (e.g., “kidney: cortex”) on the left is linked to anatomically correct 3D reference objects in the middle. A listing of cell types commonly found inside these anatomical structures can be seen in the lower left. The data for these listings come from the Biological Structure and Spatial Ontologies in the CCF-HRA. A list of registered tissue blocks corresponding to the selected anatomical structures is displayed on the right, with white 3D blocks placed within the reference organs to indicate the sizes, positions, and rotations of those tissue blocks. The CCF-HRA Specimen Ontology defines filters at the top left of the user interface for patient demographics, assay types, and tissue providers.

### Usage of the RUI and EUI

The CCF-HRA metadata and associated 3D Reference Object Library are unique in that they interlink specimen, biological structure, and spatial data in support of HRA construction and usage. The RUI and EUI use the CCF-HRA to describe collected tissue blocks and biopsies in a standardized way which enables data harmonization across consortia, donors, organs, and assay types (see Fig. 3).

The RUI requires about five minutes of training time and two minutes for each tissue registration^21^. As of April 2022, 5909 tissue blocks have been collected, including 506 by HuBMAP^3^, six by HCA, and 5397 by GTEx^22,23^. 5856 tissue sections were derived from the 506 HuBMAP tissue blocks. The resulting data covers 16 organs with 12 organs featured by HuBMAP (see HRA Dashboard in Supplementary Fig. 1). While all HuBMAP RUI locations are linked to tissue block samples in the HuBMAP Data Portal, RUI locations from SPARC^24^ and KPMP^25^ point to papers; GTEx RUI locations lead the user to the GTEx data portal.

Note that there is a difference between RUI locations (modeled as ‘*extraction sets*’ in the CCF-HRA Spatial Ontology) and tissue blocks in that one RUI location can be assigned to many tissue blocks. For example, GTEx needed to define only 29 RUI locations across 13 organs to spatially register 5397 tissue blocks. RUI locations are defined via a RUI JSON file with metadata about the creator and the colliding anatomical structures as well as geometric information about the tissue block, such as 3D size, position, and rotation. An exemplary RUI JSON file is shown in Supplementary Fig. 3 and on GitHub at https://cns-iu.github.io/HRA-supporting-information/rui_registration.json.

The average size of the 506 HuBMAP tissue blocks is 13.8 mm width, 15.2 mm height, and 9.2 mm depth. Exactly 62 tissue blocks have associated scRNAseq gene biomarker data from which cell type annotations can be derived using Azimuth^26^ or similar services; 90 datasets use validated antibody panels that detect well defined protein biomarkers and the cell types those characterize—supporting cell type identification at the single-cell level and are spatially explicit, e.g., they use CODEX^6^ or Cell DIVE^27^, making it possible to run segmentation algorithms at the anatomical structure, FTU, or single-cell level. Other datasets use Imaging Mass Spectrometry^28^, Sequential Fluorescence In-Situ Hybridization (seqFISH)^29^, Slide-seq^30^, or Visium^5^. Examples of spatially explicit datasets are the six HCA heart tissue blocks^31^, the 19 RUI-registered kidney tissue blocks, or the 12 skin tissue blocks. GTEx^23^ is an example of having multiple datasets from the same extraction sites, as defined by their standard operating procedures (SOPs)^32^.

An additional important use case for the RUI and EUI is to evaluate the coverage and quality of the HRA to inform future tissue data acquisition and analysis. Single-cell analysis is costly (e.g., scRNAseq data costs about $1 per cell; sampling a tissue block of 1 mm^3^ filled with 10 μm diameter cells costs about $1 million). Smart data sampling is needed to arrive at data that captures not only key anatomical structures of single organs but also the diversity of healthy male and female adults. The CCF-HRA and the presented user interfaces make it possible to compare the coverage of data across projects, as illustrated in the HRA Dashboard (see Supplementary Fig. 1), which can be explored interactively at https://hubmapconsortium.github.io/hra-data-dashboard.

## Discussion

The RUI and EUI were released in May 2021 with continuous updates following. Since then, they have been adopted by six consortia contributing data toward the HRA. Both user interfaces are available as web components, which have been successfully integrated in the HuBMAP portal and rebranded for the GTEx^33^ and SenNet portals^34,35^. The CCF-HRA’s three ontologies have been fully implemented for 25 organs. They enable the RUI and EUI to capture (1) specimen metadata about the donor (the “who”); (2) biological structure data, which describes “what” part of the body a tissue sample came from and details AS, CT, and biomarkers of the tissue sample; and (3) spatial data, which indicates “where” a tissue sample is located in a shared 3D common coordinate system.

### Limitations

Known limitations for the RUI include a limited set of reference organs (25 currently) with comparatively large anatomical structures and a not yet adressed need to  scale tissue blocks based on size differences between the donor organ and the reference organ. In addition, manipulating the 3D position and rotation of virtual tissue blocks in a 3D scene inside a 2D browser can be challenging, especially for users with little or no experience with 3D tools. However, recent work has shown that new RUI users can register a tissue block, taking on average 22.6 seconds per block and achieving a 5.9 degrees rotation and 1.32 mm position accuracy after around 8 tasks in the series of 30 identical tasks^21^.

Known limitations for the current EUI include limited filters and no deep linking capabilities. We run informal user studies for 3D spatial search and this new functionality became available in July 2022. Long loading times due to the increasing number of 3D reference organs and registered tissue blocks have been addressed in a recent release of the EUI that decreases loading times by a factor of 10.

While the CCF-HRA is a work-in-progress, the current data structures and user interfaces demonstrate the entire workflow from human tissue acquisition and registration via the RUI to data representation and exploration within the EUI for a rigorously defined and standardized data registration and management process. We are sharing this first CCF-HRA implementation to build awareness of our work and to obtain feedback and suggestions from the broader community, including other efforts that aim to map the human body at single-cell resolution. In addition, we seek feedback from scientists who are interested in using HRA data for their research, integrating CCF-HRA user interfaces into their workflows, and/or incorporating CCF-HRA data structures and user interfaces into their portals.

### Next steps

The ultimate goal of this effort is to develop effective means to interlink and federate new and existing data across scales (from anatomical structures, to cell types to biomarkers) and to link the evolving HRA to existing ontologies and 3D references. This will make it possible to understand and communicate the spatial organization of the human body across scales; interpret new datasets in the context of the evolving CCF-HRA (e.g., support cell type label transfer as done by Azimuth^26^); and map tissue blocks spatially based on their unique morphology and molecular “fingerprint” (e.g., given scRNA-seq data for a new tissue block, this block can be mapped into the 3D space that has a similar cell-type population with comparable biomarker expression values).

New datasets, technologies, and user requirements will both demand and make possible continuous improvements of the 3D reference object library and ontologies, the registration, mapping, and exploration processes, and the user interfaces. The CCF-HRA and associated user interfaces are expected to evolve to support ever more robust and detailed registration and exploration of semantically and spatially annotated tissue data.

Developing a CCF-HRA for the human body is a major undertaking that requires access not only to high-quality and high-coverage data but also to human expertise across both biological and technological domains. It seems highly desirable to develop and agree on data formats across consortia and to develop data infrastructures and user interfaces that empower many users to contribute to the construction and usage of a HRA. Serving as a “rosetta stone,” the ASCT+B tables help translate and federate data across organs and let many experts contribute. A user interface like the RUI makes it possible to register tissue samples spatially and semantically in a uniform manner in support of cross-consortium exploration via the EUI.

Experts interested in joining this international effort are invited to register via the survey at https://iu.co1.qualtrics.com/jfe/form/SV_bpaBhIr8XfdiNRH to receive regular updates and invites to relevant meetings that aim to advance the construction and usage of an HRA.

## Methods

### CCF-HRA Specimen Ontology and data

CCF-HRA Specimen Ontology describes the demographic, clinical, and other metadata associated with human specimen tissue samples. The complete HuBMAP clinical data—covering more than 100 metadata fields—was reduced to a smaller set of metadata fields that are relevant for CCF-HRA construction and usage. CCF-HRA specimen data was then compared with data made available by other consortia to finalize data fields relevant for CCF-HRA design. The current CCF-HRA specimen data includes demographics and clinical data (e.g., sex, age, body mass index [BMI]), workflow information (e.g., tissue sample creation/modification date, donor/organ/tissue ID, specimen/data/assay type), author information (e.g., author group/creator), and links to source data/publications. Additional components of the CCF-HRA specimen data link samples to the laboratories (tissue mapping centers [TMCs] in HuBMAP) and projects that collected the tissue, and assay types (e.g., CODEX, Periodic acid–Schiff, or autofluorescence). Note that for HuBMAP data, specimen information is created on the fly by querying the HuBMAP search-API. For data from other consortia, specimen information is stored in the *rui_locations.jsonld* file for each tissue block.

The complete set of specimen data consists of data collected during data ingestion and data generated during tissue registration using the RUI. Data is shared in JSON with the following 22 fields: donor ID (de-identified), age (or age range), sex, BMI (kg/m^2^), consortium name, tissue provider name, tissue provider UUID, tissue provider author, links to source data, tissue block ID, tissue block dimensions (width, height, depth, and unit of measurement), tissue block position (called translation) and tissue block rotation (both relative to the 3D space shared with the CCF-HRA reference organ), tissue block placement author, number of tissue sections (for a given tissue block), size of tissue sections (including units of measurement), tissue section ID, tissue section number, dataset ID, dataset technology used, dataset assay type, and dataset thumbnail.

### CCF-HRA Biological Structure Ontology and data

In addition to registration and exploration user interfaces, the user requirements assessment identified the need for agreement across multiple consortia and stakeholders on what anatomical structures, cell types, and biomarkers are relevant for construction and usage of an HRA. The biomarkers include cell type-specific genes, proteins, lipids, and metabolites commonly used to characterize cell types that are located in specific anatomical structures. Collectively, we call this the ASCT+B terminology. Furthermore, anatomical structures and cells exist in a 3D context, and there is a need for 3D representations of major anatomical structures and cell types so that experimental data can be spatially registered, explored, and analyzed in the context of this 3D HRA.

**Fig. 4:**
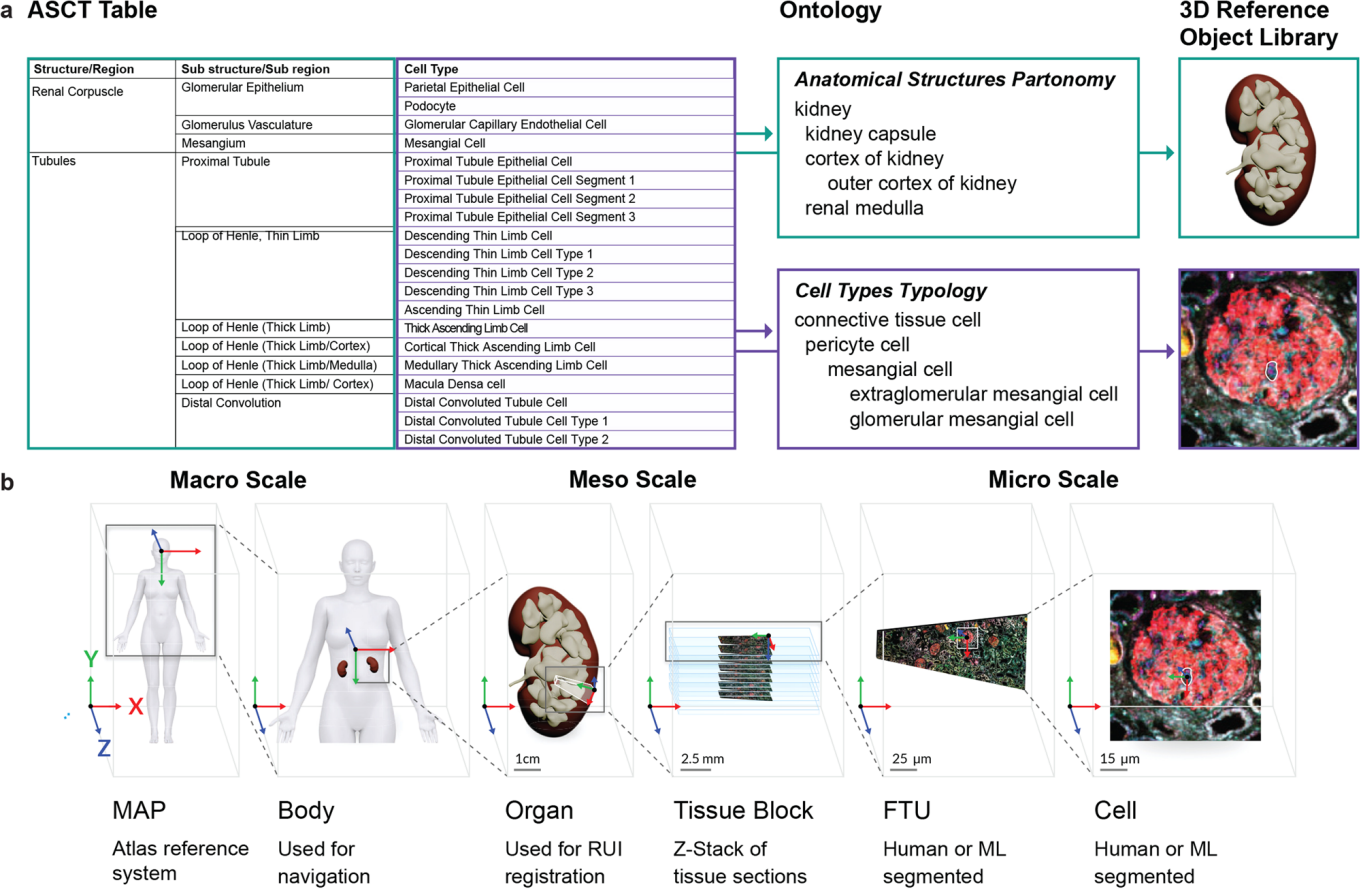
Semantic and Spatial Representation of a Kidney. **a** The CCF Biological Structure Ontology represents the body as a set of nested, named anatomical structures (the anatomical structures “partonomy”) and cell types (the cell type “typology”). Anatomical structures and cell types are linked to Uberon/FMA and CL ontologies respectively, and are mapped to 3D reference objects at macro to single-cell level. **b** The CCF Spatial Ontology leverages the 3D Reference Object Library to define the dimensions and shapes of ASCT+B entities within the HRA 3D space (called the ‘Atlas reference system’). The male and female VHP reference bodies are registered within the HRA space. All organ reference objects are registered to the respective male or female VHP reference body. A new tissue block is sized, positioned, and rotated relative to a reference organ. Segmentation masks for single cells and functional tissue units identified via manual or automatic means are spatially registered with their respective tissue section images–z-stacks of 2D tissue sections make up 3D tissue blocks.

A parallel effort by 17 consortia^19^ is therefore creating (1) ASCT+B data tables that capture major anatomical structures, cell types, and biomarkers and their interrelationships—including *part_of* relations (the hierarchical “partonomy”) between anatomical structures, *located_in* relations between cell types and anatomical structures, and *characterizes* relationships between biomarkers and cell types, and (2) associated 3D reference objects that represent the size, position, rotation, and shape of major anatomical structures that are listed in the ASCT+B tables. While organs differ substantially in their form and function and across human specimens, all have anatomical structures, cell types, and biomarkers that are captured in the ASCT+B tables and the associated 3D Reference Object Library^36^. The resulting ASCT+B tables represent expert knowledge and information from textbooks, scholarly publications, and experimental data evidence. An associated 3D Reference Object Library captures the anatomically correct 3D shapes, sizes, locations, and rotations of key anatomical structures present in the ASCT+B tables. The organs are developed by specialist 3D medical illustrators, and they are approved by organ experts^37^. Please see Fig. 4a for an exemplary partial ASCT+B table and corresponding 3D reference objects of a kidney.

The SOP for ASCT+B table authoring details this process^38^. In short, anatomical structure, cell type, and biomarker names are compiled with their unique identifiers in existing ontologies, such as Foundational Model of Anatomy (FMA)^39–41^, Uber-anatomy ontology (Uberon)^42^, Cell Ontology (CL)^43^, and Human Genome Nomenclature Committee (HGNC)^44^ in machine readable, computable format. Note that the existing ontologies have tens of thousands of terms, many of which are not relevant for healthy human adults (e.g., terms for capturing development and growth, cross-species comparisons, and disease). By authoring the ASCT+B tables, we have discovered omissions in existing ontologies that need to be resolved to properly represent healthy human adults. In these cases, we are working closely with the respective ontology curators to add these terms and corresponding literature justifications to the ontologies so they properly represent the HRA. The resulting CCF-HRA Biological Structure Ontology exclusively captures terms relevant for healthy human adults. An ASCT+B Table Reporter website was built to visualize the CCF-HRA biological structure data (Supplementary Fig. 2). It facilitates the construction and review of the ASCT+B tables by domain experts and enables new users of the RUI and EUI to explore and become familiar with the data.

In December 2021, 25 ASCT+B tables and 50 associated reference organs were published, Digital Object Identifiers (DOIs) were minted, and all files were made publicly available^45^. In addition, the ASCT+B tables were published using Semantic Web technologies in Web Ontology Language (OWL 2)^46^. The CCF.OWL was deposited in BioPortal^47^ and the EMBL-EBI Ontology Lookup Service (OLS) [cite https://www.ebi.ac.uk/ols/ontologies/ccf], both support version control. Counts of the number of entities and relationships for the 25 organs can be found in Supplementary Table 1. For example, the ASCT+B for the kidney features 61 anatomical structures, 62 cell types, and 150 biomarkers, but also 62 *part_of* relationships between these anatomical structures, 60 *located_in* relationships from cell types to anatomical structures, and 257 *characterize* relationships from biomarkers to cell types.

Using the CCF.OWL, the 3D reference objects of the HRA (but also tissue blocks registered into them) are compatible with and can be linked to other ontologies and the data they describe. For example, anatomical structures or genes can be linked to chemical compounds in the ChEMBL^48–50^, Chemical Entities of Biological Interest (ChEBI)^51,52^ or diseases using the Disease Ontology^53^ and Human Phenotype Ontology (HPO)^54–58^.

### CCF-HRA Spatial Ontology and data

CCF-HRA spatial Ontology describes the 2D and 3D shapes of entities and their physical size, shape, location, and rotation—from the whole-body level down to single cells (Fig. 4b). There are three key concepts to make this work: (1) A *Spatial Entity* represents a real-world object (e.g., a human body, a human kidney, a tissue block or section, or an individual cell). It defines a bounded Cartesian space and its unit of measurement. By using the *ccf_representation_of* property, we say that a *Spatial Entity* is representing/standing in for either an ASCT+B term in the CCF-HRA Biological Structure Ontology or a physical object, such as a tissue sample. *Spatial Entities* connect to ASCT+B terms using either *ccf_representation_of* or *ccf_annotation*. (2) A *Spatial Object Reference* points to an object in the CCF-HRA Reference Object Library^36^. It provides a reference to an external representation of a *Spatial Entity*, such as a 3D object file (e.g., in OBJ, FBX, GLTF format) or a 2D image (e.g., in TIFF, PNG, SVG format). Initially, most of the anatomically correct 3D reference organs in this library are created using male and female data from the Visible Human Project (VHP) made available by the National Library of Medicine^59–61^. (3) A *Spatial Placement* defines how to place a *Spatial Entity* or *Spatial Object Reference* relative to another *Spatial Entity* using scaling, rotation, and translation (in that order). Note that rotation (in x, y, z order) occurs around the center of the object’s coordinate space; by default, rotation is considered in Euler order. In the case of *Spatial Object References*, it defines how to transform a 2D or 3D object so that it fits the *Spatial Entity*’s dimensions and unit of measurement. In the case of *Spatial Entities*, it shows how to place one *Spatial Entity* relative to another.

A unique feature of the CCF-HRA is the interlinkage of the ASCT+B to the 3D Reference Organs. A Crosswalk table^62^ links anatomical structures in the ASCT+B tables to anatomical structures in the 3D organ file. Given this interlinkage of ontology terms and 3D nested objects, new forms of semantic and spatial queries can be supported. For example, given a registered tissue block, the 3D anatomical structures it contains/collides with can be determined and used as annotations; using these annotations, all smaller anatomical structures and cell types inside of larger, colliding anatomical structures can be retrieved. The crosswalked data also makes it possible to use cell types in search queries and to retrieve all anatomical structures that typically contain a set of cell types. Plus, it makes it possible to query for and display all cell types that are in a given set of selected anatomical structures or tissue blocks, see listing of unique cell types in lower left of Fig. 2.

3D object scene graphs are created using spatial entities, spatial object references, and spatial placements. Spatial entities and spatial object references are placed relative to other spatial entities to create a 3D scene graph for use in the RUI and EUI.

In addition to the 3D Reference Objects, 3D spatial entities are created for extraction sites and tissue blocks. In general, each tissue block has a well-defined extraction site in relation to a well-defined 3D Reference Object. In addition, we capture segmentation masks for FTUs for 2D tissue sections using a GeoJSON data format.

#### 3D extraction sites and landmarks

In support of systematic tissue extraction across human donors, many laboratories/projects sample tissue using well defined “extraction sites.” Each extraction site defines the size, location, and rotation of a tissue block in relation to a reference organ, along with a set of anatomical structure annotations derived via collision detection (see sample in Fig. 1a). Given a new tissue sample, a previously defined extraction site can be used as metadata. The RUI makes it easy to define an extraction site. For example, for the heart, 6 extraction sites have been identified for HCA, 10 for HuBMAP, 25 for SPARC (see Fig. 1d), and 2 for GTEx (not shown in Fig. 1d). The process of defining extraction sites is captured in an SOP^63^. Named landmarks are available in GLB and FBX format and are saved with the female and male reference organs^64^. Further, the GLB files are semantically tagged and linked into the CCF-HRA Ontology (CCF.OWL) via the JSON-LD data.

#### 3D tissue blocks

Using the RUI, metadata can be generated for tissue blocks. All data can be inspected, corrected, and downloaded in JSON format (see example data file in Supplementary Fig. 3). Note that anatomical structure annotations (‘*ccf_annotations*’) are provided using Personalized Uniform Resource Locator (PURL) links to Uberon terms. ‘*Scaling*’ indicates how much to shrink or expand a tissue block and is defined as a ratio. ‘*Translation*’ captures how to translate a *SpatialEntity* (e.g., a tissue block) so that it is placed relative to another *SpatialEntity* (e.g., a reference organ) and it is rounded to 1 micrometer precision. In some cases, tissue sections are cut from a tissue block. RUI users can enter information on tissue section thickness under ‘*slice_thickness*,’ which is given in micrometers. The number of RUI locations publicly available for HuBMAP tissue blocks can be accessed in the HRA Dashboard (Supplementary Fig. 1).

#### 3D segmentation masks

We distinguish three types of annotated segmentation masks: anatomical structure, functional tissue unit (FTU), and single-cell masks. Exemplary masks were compiled for and used in the “HuBMAP - Hacking the Kidney” Kaggle competition^65^. The anatomical structures and FTU masks are modified GeoJSON files that capture the outline of annotations by their pixel coordinates, and they were generated from annotations by subject matter experts using QuPath^66^. Supplementary Fig. 4 shows a GeoJSON file that describes one glomerulus mask for a kidney tissue section. The yellow highlighted part lists the 15 x, y location pairs of the 14 line segments that comprise the polyline which outlines the one glomerulus shown on the right.

#### Biological structure annotation via collision detection

When a tissue sample, such as a kidney tissue block, is registered and annotated through the RUI, it is assigned a unique identifier (e.g., “UUID-S-5678”) using the CCF-HRA Specimen Ontology. It is linked to a term in the CCF-HRA Biological Structure Ontology, indicating the anatomical structures or cell type (e.g., “kidney cortex”). The CCF-HRA Biological Structure Ontology’s anatomical structure partonomy details how the sample fits within larger structures, up to the whole body. Using the CCF-HRA Spatial Ontology, samples are linked to a *Spatial Entity* (e.g., “UUID-SE-9123”), which gives its size/dimensions. A *Spatial Placement* (e.g., “UUID-SP-4567”) positions the sample relative to another *Spatial Entity* (e.g., “#VHKidneyVH_M_right_kidney”). After proper registration, all colliding objects are identified and corresponding anatomical structure terms from the ASCT+B tables are assigned to the tissue block as “*ccf_annotations*.” Further, experts are able to verify and add or remove anatomical structure terms while registering.

### Reporting summary

Further information on research design is available in the Nature Research Reporting Summary linked to this article.

## Supplementary information

Supplementary Information

Reporting Summary

## Data Availability

All data are open and free to access. The ASCT+B tables and associated 3D reference objects for 25 organs published in the second CCF-HRA release v1.1 are available at the CCF-HRA Portal, https://hubmapconsortium.github.io/ccf. Registration data for HuBMAP, SPARC, HCA, KPMP, and GTEx are available at https://portal.hubmapconsortium.org/ccf-eui. For HuBMAP data, tissue blocks link to sample and donor metadata and raw data. For all other data, the blocks link to relevant papers or datasets in the SPARC, HCA, KPMP, and GTEx data portals. All existing ASCT+B tables can be downloaded from https://hubmapconsortium.github.io/ccf/pages/ccf-anatomical-structures.html. Organs in the CCF-HRA 3D Reference Object Library are available as GLB files at https://hubmapconsortium.github.io/ccf/pages/ccf-3d-reference-library.html. Further, these GLB files are semantically tagged and linked into the CCF-HRA Ontology (CCF.OWL) via the JSON-LD data format.

## Code availability

The registration and exploration user interfaces, standard operating procedures, and video demos of proper usage of both user interfaces are available via the CCF-HRA Portal, https://hubmapconsortium.github.io/ccf.

Code is publicly hosted at GitHub https://github.com/hubmapconsortium/ccf-ui. The Python script used to compute sizes of the 506 HuBMAP tissue blocks is at https://github.com/cns-iu/HRA-supporting-information.

The RUI is available via the HuBMAP Ingest Portal at https://ingest.hubmapconsortium.org/ and as a stand-alone^67^ web application. There is also an SOP for the RUI^20^.

Standard operating procedures, demonstrations, learning modules, and self quizzes exist in the Visible Human MOOC at https://expand.iu.edu/browse/sice/cns/courses/hubmap-visible-human-mooc.

The CCF-HRA v1.6 source code repository is available at http://purl.org/ccf/source. The CCF-HRA v1.6 consists of three main parts: (1) The *CCF-HRA Core Model* is an architecture for modeling 2D and 3D spatial data annotated with ontology terms. (2) The *CCF-HRA Spatial Reference System* is a curated and annotated instantiation of the Core Model covering the entire scale from the human body down to the single-cell level. (3) The *CCF-HRA Anatomical Structures Partonomy* is a curated partonomy using existing ontology terms which mirrors the CCF-HRA Spatial Reference System.

The CCF-HRA ontology has been defined as a formal ontology. Data are added as RDF/XML or JSON-LD that is translated to RDF/XML.
