# Supplementary material for: Tissue registration and exploration user interfaces in support of a human reference atlas
Source: Commun Biol. 2022 Dec 13;5:1369. doi: 10.1038/s42003-022-03644-x (PMC9747802; doi:10.1038/s42003-022-03644-x)

Supplementary Information for “Tissue Registration and Exploration User Interfaces in support of a Human Reference Atlas”

Katy Börner[1*], Andreas Bueckle[1*], Bruce W. Herr II[1], Leonard E. Cross[1], Ellen M. Quardokus[1], Elizabeth G. Record[1], Yingnan Ju[1], Jonathan C. Silverstein[2], Kristen M. Browne[3], Sanjay Jain[4], Clive H. Wasserfall[5], Marda L. Jorgensen[5], Jeffrey M. Spraggins[6], N. Heath Patterson[7], Griffin M. Weber[8]

[1] Department of Intelligent Systems Engineering, Luddy School of Informatics, Computing, and Engineering, Indiana University, Bloomington, Indiana, USA
[2] Department of Biomedical Informatics, University of Pittsburgh School of Medicine, Pittsburgh, Pennsylvania, USA
[3] Bioinformatics and Computational Biosciences Branch, Office of Cyber Infrastructure and Computational Biology, National Institute of Allergy and Infectious Diseases, National Institutes of Health, Bethesda, Maryland, USA
[4] Department of Medicine; Department of Pathology & Immunology; Department of Pediatrics Washington University School of Medicine, Saint Louis, Missouri, USA
[5] Departments of Pathology and Pediatrics, University of Florida, Gainesville, Florida, USA
[6] Department of Cell and Developmental Biology, Mass Spectrometry Research Center, Vanderbilt University School of Medicine, Nashville, Tennessee, USA
[7] Department of Biochemistry, Mass Spectrometry Research Center, Vanderbilt University School of Medicine, Nashville, Tennessee, USA
[8] Department of Biomedical Informatics, Harvard Medical School, Boston, Massachusetts, USA

*Corresponding authors
Katy Börner katy@indiana.edu
Andreas Bueckle abueckle@iu.edu

Table of Contents

Supplementary Figure 1. HRA Dashboard
Supplementary Figure 2. Visualizing ASCT+B Table Data
Supplementary Table 1. ASCT+B Table Counts
Supplementary Figure 3. Registration User Interface Metadata
Supplementary Figure 4. Tissue Segmentation Mask Data

Supplementary Figure 1. HRA Dashboard

Bar graphs with number of tissue blocks, tissue sections, and RUI locations per organ and tissue provider for HuBMAP data. An interactive version of this dashboard for all public HuBMAP data can be explored at https://hubmapconsortium.github.io/hra-data-dashboard. Note that EUI counts are higher as they include data by HCA, GTEx, and other non-HuBMAP consortia.

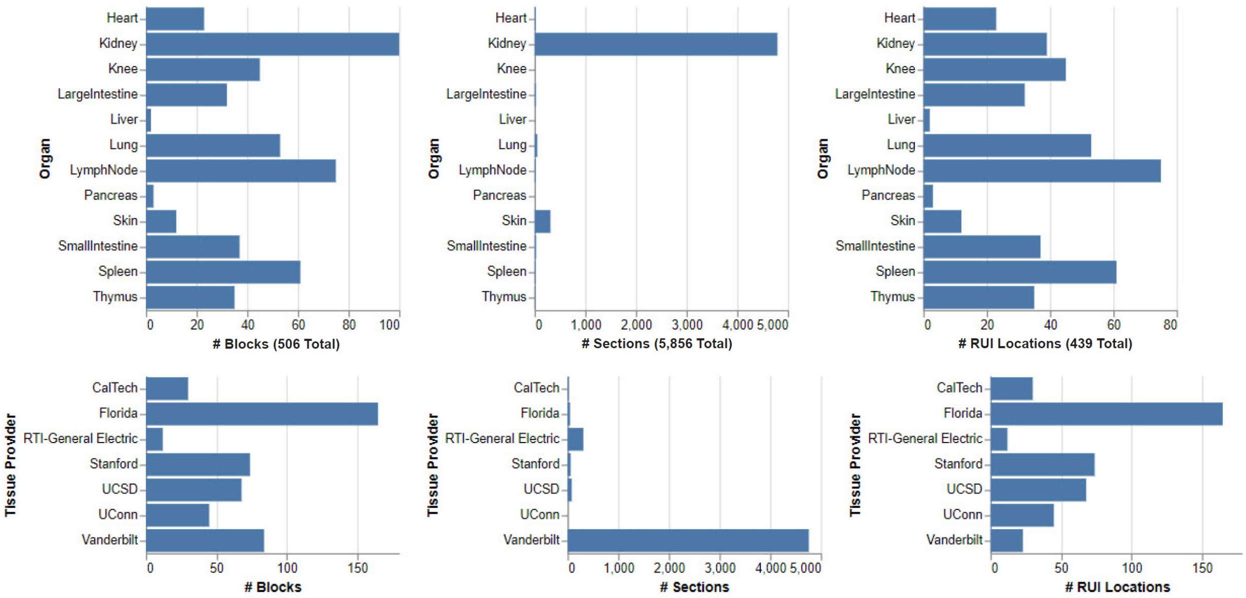

Supplementary Figure 2. Visualizing ASCT+B Table Data

The kidney ASCT+B table captures anatomical structures (red nodes), cell types (blue), and biomarkers (green) and their interlinkages. Users can hover over a node to see details and other nodes linked to it.

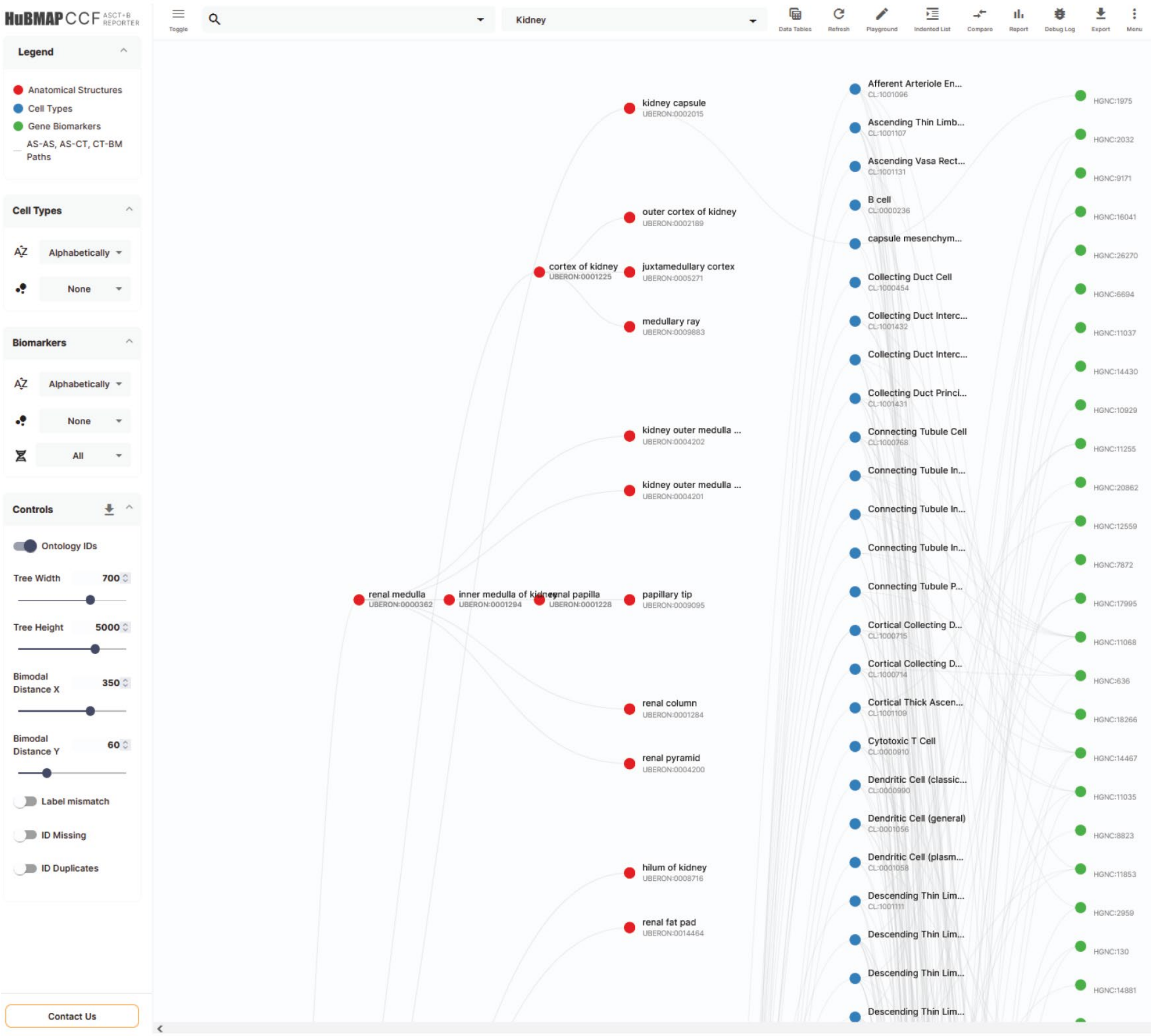

Supplementary Table 1. ASCT+B Table Counts

Counts for anatomical structures (AS), cell-types (CT), and biomarkers (B) and their relationships for the 25 ASCT+B tables published in December 2021. BG is the abbreviation used for gene biomarkers (e.g., detected using RNA sequencing) while BP refers to protein biomarkers.

Organ	#AS	#CT	#B Total	#BG	#BP	#AS-AS	#AS-CT	#CT-B
Blood	1	30	159	112	47	1	30	506
Blood Vasculature	841	2	1	1	0	869	606	2
Bone Marrow	1	47	262	198	64	1	47	838
Brain	183	127	257	257	0	183	127	346
Eye	26	53	136	61	75	27	58	404
Fallopian Tube	55	22	25	13	12	72	65	32
Heart	50	23	45	45	0	60	183	74
Kidney	61	62	150	150	0	62	60	257
Knee	32	19	14	0	14	32	8	17
Large Intestine	54	57	167	84	83	287	1,156	352
Liver	17	30	62	16	46	17	31	75
Lung	146	83	180	174	6	909	1065	267
Lymph Node	34	45	223	106	117	43	86	499
Lymph Vasculature	4	1	1	1	0	4	2	1
Ovary	71	7	13	7	6	109	12	5
Pancreas	32	32	44	42	2	162	229	101
Peripheral Nervous System	782	1	2	1	1	803	609	2
Prostate	4	12	31	31	0	4	12	36
Skin	15	36	70	0	70	17	19	101
Small Intestine	38	48	13	13	0	69	185	13
Spleen	37	61	194	85	109	50	129	424
Thymus	17	52	394	318	76	28	39	620
Ureter	7	14	30	30	0	7	14	61

Urinary Bladder	16	15	30	30	0	16	16	63
Uterus	58	19	45	39	6	73	28	65
**Totals:**	**2,582**	**898**	**2,548**	**1,814**	**734**	**3,905**	**4,816**	**5,161**

Supplementary Figure 3. Registration User Interface Metadata

Exemplary JSON file with metadata for one tissue block registered in the left male kidney reference organ (left) and corresponding RUI user interface elements (right). Tissue block dimensions are entered by users via RUI and captured in JSON file–as well as tissue block location, rotation but also six anatomical structure tags (see Uberon URLs in JSON). The JSON file can be explored in the Supporting Information website at https://cns-iu.github.io/HRA-supporting-information/rui_registration.json.


```
{
  "@context": "https://hubmapconsortium.github.io/hubmap-ontology/ccf-context.jsonld",
  "@id": "http://purl.org/ccf/1.5/bdf64f94-ccd8-484f-8eaa-a99fb9d125e8",
  "@type": "SpatialEntity",
  "creator": "Jane Doe",
  "creator_first_name": "Jane",
  "creator_last_name": "Doe",
  "creation_date": "2022-04-20",
  "ccf_annotations": [
    "http://purl.obolibrary.org/obo/UBERON_0002015",
    "http://purl.obolibrary.org/obo/UBERON_0000362",
    "http://purl.obolibrary.org/obo/UBERON_0004200",
    "http://purl.obolibrary.org/obo/UBERON_0001225",
    "http://purl.obolibrary.org/obo/UBERON_0001284",
    "http://purl.obolibrary.org/obo/UBERON_0002189"
  ],
  "x_dimension": 10,
  "y_dimension": 12,
  "z_dimension": 15,
  "dimension_units": "millimeter",
  "placement": {
    "@context": "https://hubmapconsortium.github.io/hubmap-ontology/ccf-context.jsonld",
    "@id": "http://purl.org/ccf/1.5/bdf64f94-ccd8-484f-8eaa-a99fb9d125e8_placement",
    "@type": "SpatialPlacement",
    "target": "http://purl.org/ccf/latest/ccf.owl#VHMLeftKidney",
    "placement_date": "2022-04-20",
    "x_scaling": 1,
    "y_scaling": 1,
    "z_scaling": 1,
    "scaling_units": "ratio",
    "x_rotation": -18,
    "y_rotation": 82,
    "z_rotation": 0,
    "rotation_order": "XYZ",
    "rotation_units": "degree",
    "x_translation": 26.286,
    "y_translation": 87.027,
    "z_translation": 41.215,
    "translation_units": "millimeter"
  }
}
```


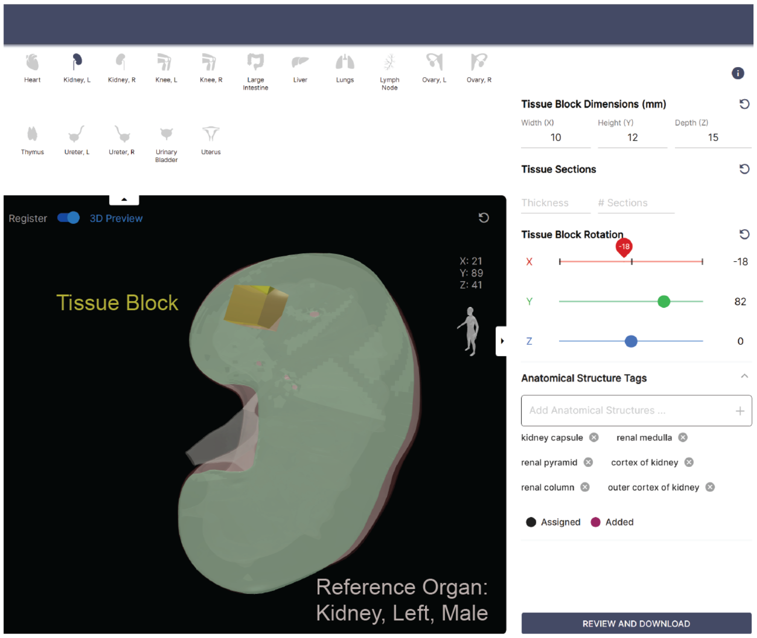

Supplementary Figure 4. Tissue Segmentation Mask Data

Exemplary JSON file for a segmentation mask for a kidney FTU on left. Kidney whole slide image (scale bar: 2mm) with zoom into one glomerulus FTU annotation on right (scale bar: 50µm). The segmentation mask consists of a 14-segment polyline. The x-y coordinates for the 15 points are highlighted in yellow in the JSON file. Note that the first set of coordinates is identical to the last–closing the polyline.


```
{
  "type": "Feature",
  "id": "PathAnnotationObject",
  "geometry": {
    "type": "Polygon",
    "coordinates": [
      [
        [26499, 2968],
        [26459, 2976],
        [26427, 3007],
        [26413, 3047],
        [26414, 3099],
        [26436, 3141],
        [26468, 3171],
        [26522, 3193],
        [26567, 3193],
        [26601, 3177],
        [26625, 3120],
        [26623, 3078],
        [26601, 3024],
        [26543, 2976],
        [26499, 2968]
      ]
    ]
  },
  "properties": {
    "classification": {
      "name": "glomerulus",
      "colorRGB": -2315298
    },
    "isLocked": false,
    "measurements": [
      {
        "name": "detection_source",
        "value": 0.995274
      }
    ]
  }
}
```


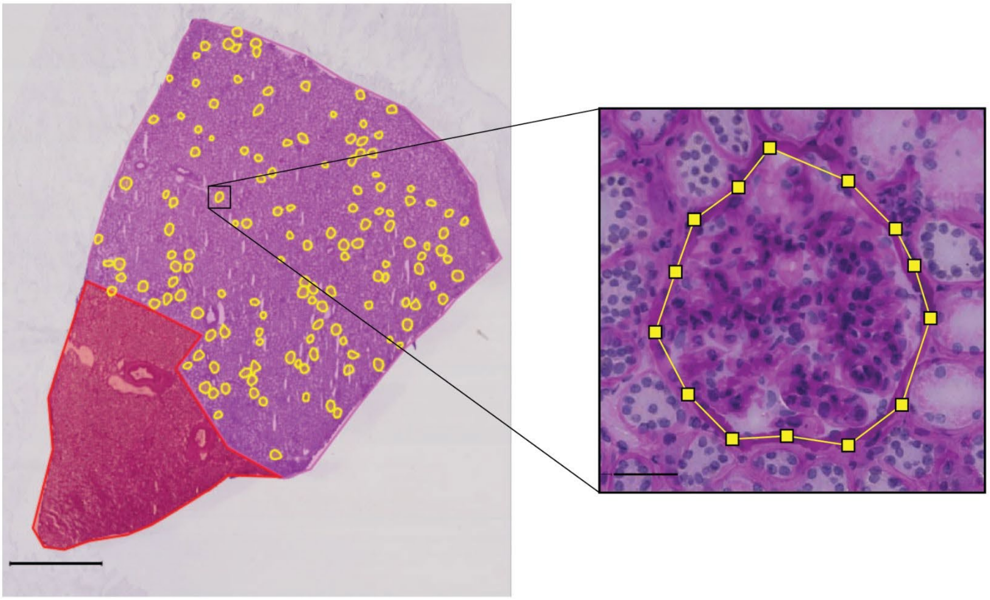

Supplement: Supplementary file 1 — Supplementary Information [file 42003_2022_3644_MOESM1_ESM.pdf]
